# Optimized workflow for digitalized FISH analysis in pathology

**DOI:** 10.1186/s13000-021-01103-5

**Published:** 2021-05-11

**Authors:** Vira Chea, Valerie Pleiner, Viviane Schweizer, Benjamin Herzog, Beata Bode, Marianne Tinguely

**Affiliations:** 1 Institute of Pathology Enge, Hardturmstr. 133, CH-8055 Zurich, Switzerland; 2 County Hospital Thurgau, Frauenfeld, Switzerland

**Keywords:** Digital pathology, Image analysis, FISH, Lymphoma, Sarcoma

## Abstract

**Background:**

Effective workflow management in a diagnostic pathology laboratory is critical to achieve rapid turnover while maintaining high quality. Fluorescence in situ hybridization analysis (FISH) is the preferred technique for detecting single chromosomal aberrations in diagnostic surgical pathology.

**Material and methods:**

FISH analysis applying a rapid hybridization protocol and using an automated whole-slide fluorescence scanning device *(3DHISTECH, Sysmex, Switzerland)* were implemented in our workflow. By analyzing 42 diagnostic cases, effects of two different scanning profiles on scanning time, and device memory usage were investigated. Manual signal counting (CaseViewer) and software based signal counting *(FISHQuant)* were compared.

**Results:**

The two scanning profiles, both including a Z-stack function, differed in their exposure time and digital gain. The “low profile” setting (LP) resulted in a significantly shorter scanning time and lower storage volume compared to the “high profile” (HP) setting, making the LP ideal for routine applications. Both signal counting methods (manual versus software based) provided similar cut-offs on a test-cohort of 13 samples.

**Conclusion:**

Scanning FISH slides provides good picture quality, reduces the analysis time and allows easy picture archiving and facilitates remote diagnostics, allowing an effective workflow.

## Introduction

Interphase fluorescence in situ hybridization (FISH) has gained importance as diagnostic and predictive examination in pathology [1]. Together with its cost effectiveness, it allows for a rapid target-oriented analysis providing results within a day.

In most instances, FISH slides are analyzed by an epi-fluorescent microscope, with or without a motorized scanning platform. Signal counting is done either manually at the microscope or at a computer screen or automatically by software-supported algorithms.

Bright field whole-slide imaging (WSI) for Hematoxylin and Eosin (H&E) stained slides and immunohistochemistry is already used in many routine diagnostic laboratories. However, scanning FISH slides is not widely used yet. Our aim was to introduce the WSI for FISH slides into routine diagnostics. A further goal was to accelerate and standardize the FISH analysis process by introducing a rapid hybridization protocol and an automated cell-signal counting program.

Herein, we report our experiences in establishing an optimal workflow for our digitalized FISH technique. Furthermore, we discuss the pros and cons of different scanning profiles and the value of an automated cell counting software.

## Materials and methods

### Pre-analytical workflow

First, a representative tumor area was encircled on the H&E-slide by a pathologist. Second, the corresponding area was marked with a diamond pen on the back of the slide to be hybridized. This narrowed down the tissue surface to be scanned since the diamond scratches remain visible on the scan-preview.

### FISH technique

A standard protocol was established for FISH on formalin fixed, paraffin embedded (FFPE) specimens. A tissue-micro-array (TMA) with ten cores (at 3mm^2^ diameter) for the probe set up and 42 diagnostic samples including core needle and excisional biopsies were analyzed. Adapted to the tissue type, the pretreatment time varied from 30 to 40 min. The specific probes (Zytovision, Germany or Vysis, Abbott Molecular, USA) were hybridized at 37 °C for 4 h in the presence of the IntelliFISH Hybridization buffer (Vysis, Abbott Molecular, USA). As nuclear stain and mounting medium the DAPI (4′,6 diamidino-2-phenylindole) VECTASHIELD® HardSet™ (Vector laboratories, CA, USA) with a minimum hardening time of 30 min was used.

### Slide imaging and analyzing

The optical system of the Pannoramic 250 Flash II Scanner (3DHISTECH, Sysmex, Switzerland) contains two Zeiss (Jena, Germany) Plan-Apochromat dry objectives (20x and 40x with a numerical aperture of 0.80 and 0.95 respectively) allowing also bright field scanning. In a motorized software-controlled wheel, three fluorescence filters are incorporated: FITC (green light, 459 nm), TRITC (red light, 544 nm) and DAPI (autofluorescence, 360 nm). The “SPECTRA light (Lumencor, USA) engine 6” switches the filters fast without fading. Images are acquired by the 16-bit scientific CMOS pco.edge 4.2 camera.

We scanned all slides with the 40x objective with a resolution of 0.25 um/pixel. To circumvent inherent tissue-quality fluctuations, two main scanning profiles (low (LP) and high (HP)) were generated. The profiles differ in their exposure time (ET) (LP: 150 ms vs HP: 2000 ms) for the FITC and TRITC channels and their digital gain (LP: 3–4 vs HP: 0–2). For both profiles, the Z-stack function was activated using five to seven layers with a layer distance of 0.4 μm. The scanning time (min), the file size (MB) and the fields of view (FOV) of the two profiles were compared (Table 1B). The area-scanning technology of the current scanner is FOV based. The FOV corresponds to the square image of the camera sensor. The larger the area to be scanned, the greater the number of the FOV required. When using different profiles with the identical area to be scanned, the number of FOV remains the same.

**Table 1:** Summaries of FISH cut-off and scanning profile

A) Break apart probes with comparison of cut off values from the literature. ° dual colour dual fusion			
**Probe**	**Provider**	**Cut off (%)**	**Cut in the literature (%)**
**BCL-2**	**Zytovision**	4	10
**BCL-6**	**Zytovision**	4	8
**CCND1**	**Zytovision**	4	1.3
**COL-A1**	**Zytovision**	5	5 °
**EWSR1**	**Vysis/Abbott**	7	10
**FUS**	**Vysis/Abbott**	3	NA
**C-MYC**	**Vysis/Abott**	5	9
**USP-6**	**Vysis/Abbott**	3	20
B) Comparison of scanning time, file size and FOV (fields of fiew) scanned with the two profiles in 42 diagnostic cases			
**Profile**	**All (*n*** **= 42)**	**High (*n*** **= 26)**	**Low (*n*** **= 16)**
**scanning time in min**			
**Min**	1	3	1
**Max**	1620	1620	72
**Mean, Total**	87	159	15
**Mean, <10’000 FOV**	34	57	11
**file size**			
**Min**	17	17	30
**Max**	7620	7620	940
**Mean, total**	794	1129	458
**Mean, <10’000 FOV**	468	607	328
**FOV**			
**Min**	105	105	120
**Max**	58830	58830	10620
**Mean, total**	3840	5971	1708
**Mean, <10’000 FOV**	1713	2311	1114

### Manual counting

Digitalized images were visualized in the CaseViewer (3DHISTEC, Sysmex, Switzerland), a digital microscope application software. As a control step, the pre-selected areas of the corresponding H&E- and FISH-slides were viewed in parallel. Thereafter, FISH signals of a hundred of nuclei were counted manually at the computer screen. Cut-off levels were assessed as described earlier [2]. A signal was counted as abnormal, when the green and the red signal were two diameters of one signal apart.

### Software counting

FISHQuant (3DHISTECH, Sysmex, Switzerland), is an IVD approved module allowing to automatically quantify structural and numerical FISH signal abnormalities in solid tumors and neoplasias of the hematopoietic system. Since automated classification is error prone due to tissue inherent artefacts like overlapping of nuclei, manual editing is mandatory before signing out final reports.

## Results

### FISH technique

**Fig. 1:**
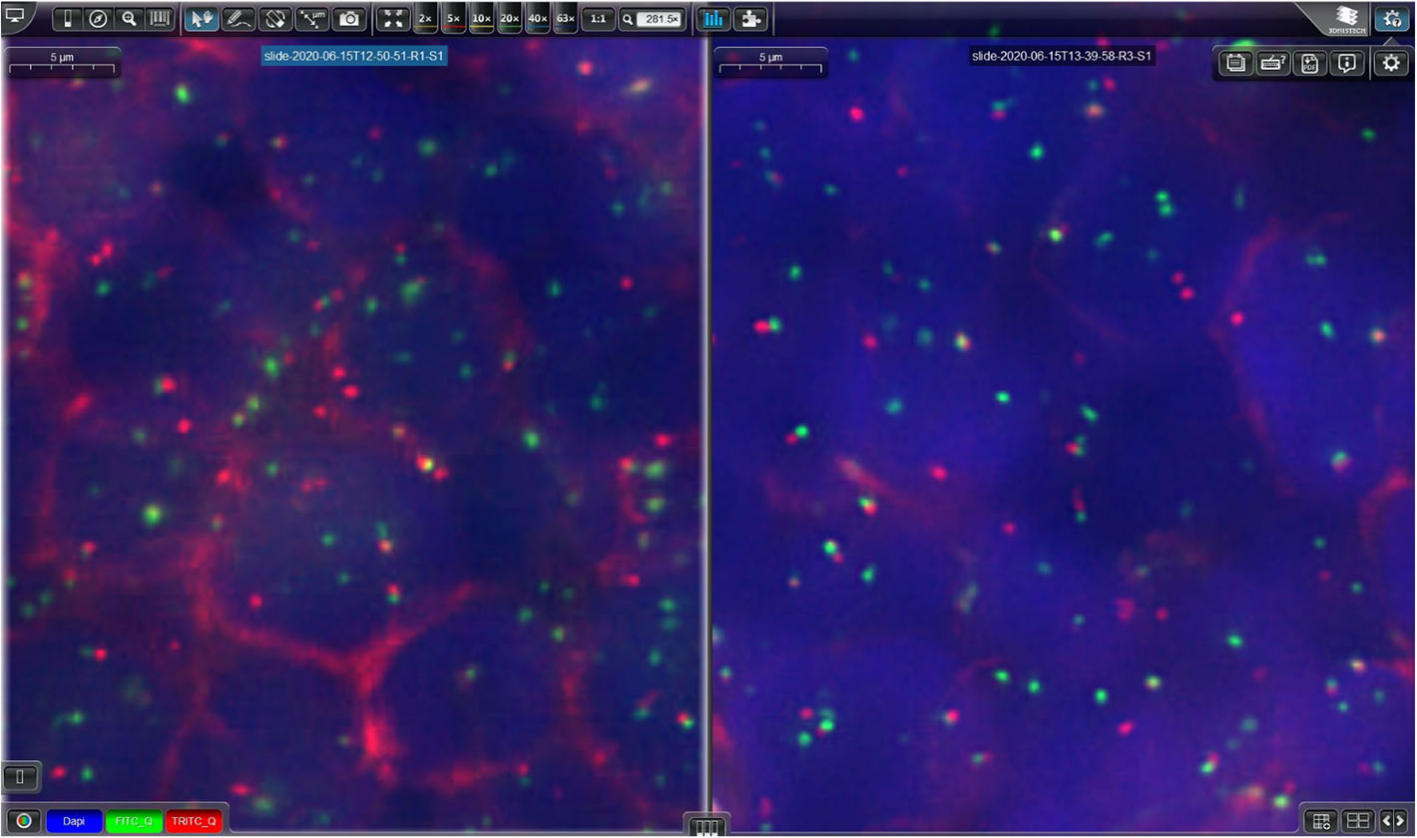
Comparison of a case scanned by the low profile (**a**) versus high profile (**b**) using a C-MYC bap probe. The high profile results in a better signal to noise ratio without fading. The settings applied are shown in (**c**) resulting in different storage sizes

**Fig. 2:**
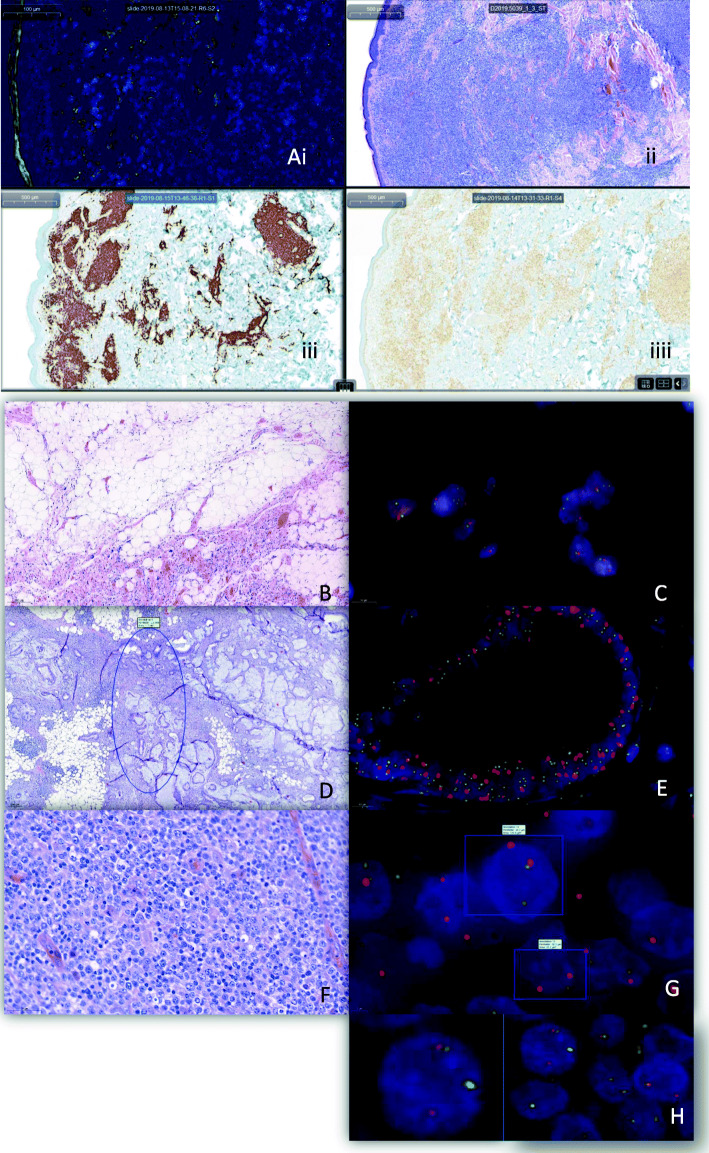
Clinical samples of routine diagnostic cases with the corresponding HE: (**a**) Parallel viewing of a primary cutaneous marginal zone lymphoma: (i) BCL2 BAP probe, (ii) HE, (iii) CD20 (iiii) Bcl2; (**b**, **c**) Lipoma without MDM2 amplification; (**d**, **e**) Metastases of a cholangiocellular carcinoma showing the pre-analytical tissue selection area annotated on the CaseViewer and the corresponding MDM2 amplification; (**f**, **g**) “double hit” lymphoma with a C-MYC break in the annotated tumour cells and a break in the BCL-6 gene (**h**)

Introducing the IntelliFISH Hybridization buffer substantially shortened the hybridization process from 18 to 4 h and resulted in good signal to noise ratios with strong and distinct signals (Figs. 1 and 2). The DAPI hardening mounting media VECTASHIELD® HardSet™ proved to be the fastest option of the several types of media tested.

### Slide imaging and analyzing

The handling of the scanner software turned out to be intuitive and rather easy. Both established profiles (LP and HP) provided signals of good quality, however, the HP translated a better signal to noise ratio (Fig. 1). The scanning time of a TMA core (3mm^2^) varied between 5 to 7 min with a LP and 15 to 20 min with a HP.

The scanning time for FISH depended on the FOV reflecting the size of the selected area and the exposure time (ET) per fluorescence channel. In 16/42 samples we applied an LP (150 ms ET) and in 26/42 samples a HP (2000 ms ET) (Table 1B). With this approach, the scanning time was more than ten times longer for the HP (mean 170 min) than for the LP (mean 15 min). Moreover, the file size was 2.5 times larger for the HP than for the LP (Table 1B) while the mean file size per FOV remained comparable for all approaches (low: 0.32; high: 0.34) as expected. In four cases (one with LP and three with HP) the FOV was > 10′000 resulting in high data volumes (min 2390 MB, max 7620 MB, mean 4453 MB) leading to the longest scanning time with the LP (72 min) and HP (949 min). Whereas the LP showed strong enough signals to be successfully analyzed in most instances, HP improved picture quality in cases with weak signals or high background (Figs. 1b and 2).

### Manual vs automated counting

In three out of the 42 diagnostic samples and the TMA, the FISHQuant software automatically classified the signals and the nuclei correctly. Compared to manual counting FISHQuant provided similar results within seconds (e.g. 7% vs 6% for ETVS1). However, in the remaining samples, especially those containing lymphatic tissue, the nuclei were too densely packed to be correctly identified by the automatic algorithm, leading to a high number of erroneously classified signals.

## Discussion

FISH has become an important theranostic auxiliary method in surgical pathology over the years [1]. To meet the current needs of shortening turn-around-times while maintaining high quality and cost-effectiveness, we accelerated the hybridization process by introducing the IntelliFISH hybridization buffer. Thereby, we shortened the duration of the experimental process by more than 12 h while preserving an excellent signal quality. The overall time from the entry of the order to the hybridized slide was cut to approximatively 6 h with around 30 min hands on time.

The Pannoramic 250 Flash II Scanner equipped with a fluorescent module has proven to be a reliable and efficient tool for routine diagnostics of break-apart and enumeration probes. Our observations are in line with a previous report regarding the same system and a second one dealing with a different scanning system [3, 4]. One main difference and advantage compared to conventional fluorescence microscopes is the lack of fading of the fluorescent probes during the scanning process. Additional major advantages of digitalizing FISH slides are the preview and the alignment of the hybridized slides with their corresponding H&E or immunohistochemical stain on the CaseViewer, allowing a more precise as well as fast, identification and analysis of the diseased area (Fig. 2) [4]. Other benefits for the examiner compared to the use of a traditional fluorescence microscope were the larger fields of view and wider zoom-ranges. Both could be easily and continuously adjusted on the CaseViewer without losing the area of interest. This simplified analysis and the optimized FISH protocols might be reasons for the lower cut-off values for our probes as compared to those described in the literature (Table 1A) [5]. However, the methods used for the assessment of the thresholds were not indicated in all reports [6–9].

Based on our experience, the establishment of two different scanning profiles is sufficient to enable a routine diagnostic FISH laboratory to easily scan and analyze tissue samples of different origin. A mean scanning time of 15 min for the majority of samples applying the LP seems reasonable. Hence, the HP can be reserved for more demanding probes.

In our hands, the automated FISHQuant software is promising and provides graphically represented results of break-apart probes within seconds. However, the ability to discriminate nuclei and to correctly assign the signals to them is limited by the algorithm, necessitating an elaborate manual editing compared to the manual counting by means of the CaseViewer. Therefore, the FISHQuant software is not yet ideal for certain tissues, especially not for lymphomatous tissue, since the algorithm is only able to correctly classify a minority of nuclei. A further refinement into a self-learning system would be desirable.

In conclusion, in our view the advantages of scanning FISH slides far outweigh the conventional analysis by fluorescence microscopes. Particularly storage, sharing and remote diagnostics open up new opportunities. The development of tissue adapted self-scoring software would be desirable.
